# ‘Read–through marking’ reveals differential nucleotide composition of read-through and truncated cDNAs in iCLIP

**DOI:** 10.12688/wellcomeopenres.14663.1

**Published:** 2018-06-22

**Authors:** Ina Huppertz, Nejc Haberman, Jernej Ule

**Affiliations:** 1 Department of Molecular Neuroscience, UCL Institute of Neurology, London, WC1N 3BG, UK; 2 European Molecular Biology Laboratory (EMBL), Heidelberg, 69117, Germany; 3 MRC London Institute of Medical Sciences, London, W12 0NN, UK; 4 The Francis Crick Institute, London, NW1 1AT, UK

**Keywords:** Protein–RNA interactions, iCLIP, High-throughput sequencing, Polypyrimidine tract binding protein 1 (PTBP1), Eukaryotic initiation factor 4A-III (eIF4A3)

## Abstract

We established a modified iCLIP protocol, called ‘read-through marking’, which facilitates the detection of cDNAs that have not been truncated upon encountering the RNA–peptide complex during reverse transcription (read-through cDNAs). A large proportion of these cDNAs would be undesirable in an iCLIP library, as it could affect the resolution of the method. To this end, we added an oligonucleotide to the 5’-end of RNA fragments—a 5’-marker—to mark the read-through cDNAs. By applying this modified iCLIP protocol to PTBP1 and eIF4A3, we found that the start sites of read-through cDNAs are enriched in adenosines, while the remaining cDNAs have a markedly different sequence content at their starts, preferentially containing thymidines. This finding in turn indicates that most of the reads in our iCLIP libraries are a product of truncation with valuable information regarding the proteins’ RNA-binding sites. Thus, cDNA start sites confidently identify a protein’s RNA-crosslink sites and we can account for the impact of read-through cDNAs by commonly adding a 5’-marker.

## Introduction

**Figure 1.**
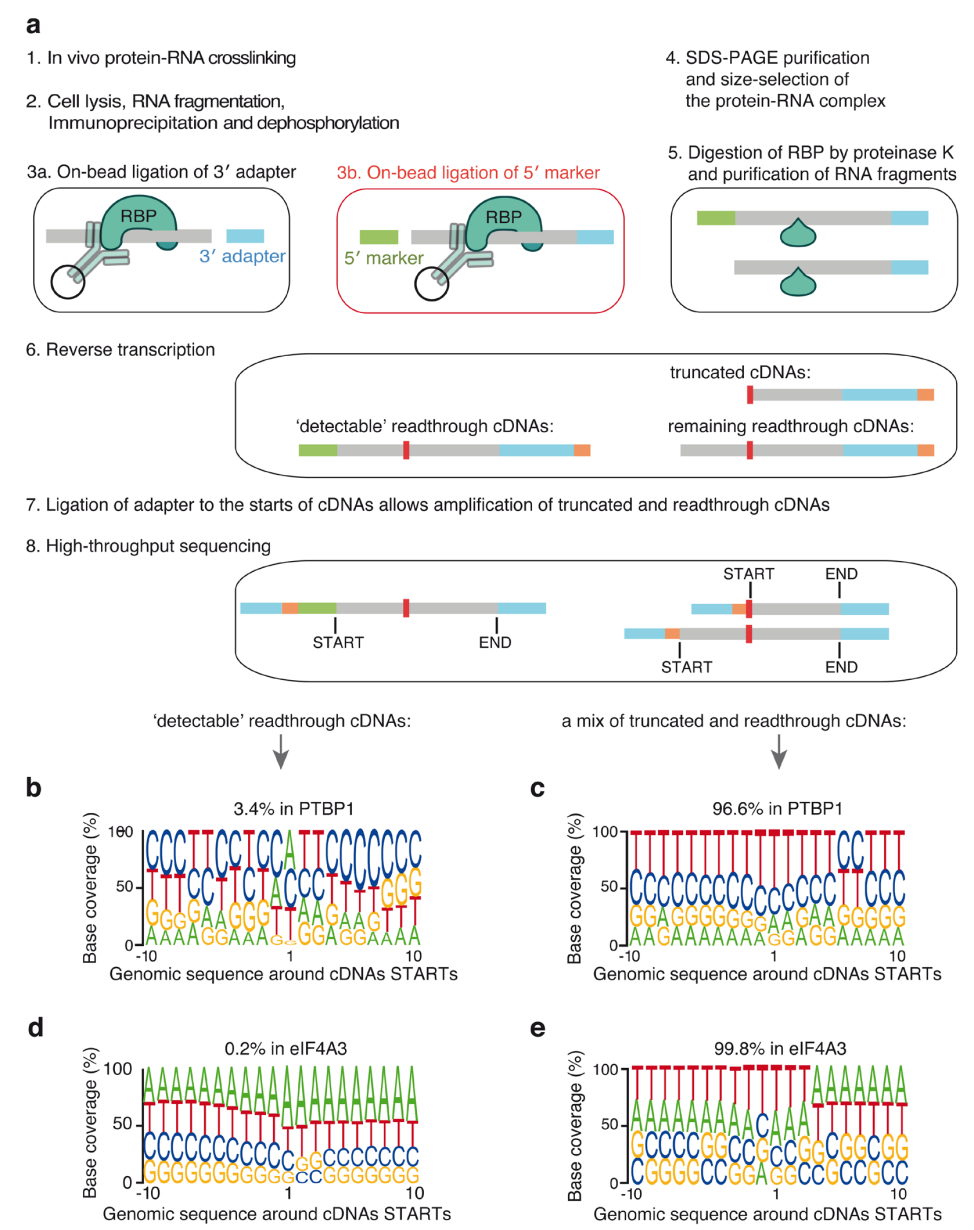
A modified iCLIP protocol identifies ‘read-through cDNAs’. ( **a**) A schematic description of the modified iCLIP protocol. Before lysis, cells or tissues are irradiated with ultraviolet (UV) light, which creates a covalent bond between proteins and RNAs that are in direct contact (step 1). After lysis, the crosslinked RNA is fragmented by a limited concentration of RNase I, and the RNA fragments are then co-immunoprecipitated with the RBP (step 2). Ligation of a 3’-adapter (step 3a) is followed by ligation of a 5’-marker that is unique to the modified protocol (red balloon, step 3b). After SDS-PAGE purification (step 4), the crosslinked RBP is removed through proteinase K digestion and purification of RNA fragments; since the ligation reaction is not 100% efficient, only a subset of the fragments contains both the 3’-adapter and the 5’-marker (step 5). Reverse transcription is performed with a primer that includes a barcode (orange) containing both an experimental identifier and a unique molecular identifier (UMI) (step 6). The peptide that remains at the crosslink site impairs reverse transcription and commonly leads to truncation of cDNAs at the crosslink site. Therefore, two types of cDNAs are generated: truncated cDNAs (which never contain the 5’-marker) and read-through cDNAs (some of which contain the 5’-marker). In iCLIP, the cDNA library is prepared so that both truncated and read-through cDNAs are amplified (step 7). After PCR amplification and sequencing (step 8), the 5’-marker sequence is present only at the beginning of read-through cDNAs. ( **b**– **e**) The composition of genomic nucleotides around iCLIP cDNA-starts that were generated using the modified iCLIP protocol; 3.4% of the mapped PTBP1 iCLIP cDNAs ( **b**) and 0.2% of the mapped eIF4A3 iCLIP cDNAs ( **d**) contained a 5’-marker (read-through cDNAs), while 96.6% of the mapped PTBP1 iCLIP cDNAs ( **c**) and 99.8% of the mapped eIF4A3 iCLIP cDNAs ( **e**) lacked the 5’-marker sequence.

As part of the individual-nucleotide resolution cross-linking and immunoprecipitation (iCLIP) protocol, crosslinked protein–RNA complexes are immunopurified and the RNA fragments are released by protein digestion, resulting in RNAs with a covalently bound peptide at the crosslink site ( König *et al*., 2010; Lee & Ule, 2018). This is followed by reverse transcription, where in theory the RNA–peptide complex leads to the premature termination of cDNA synthesis and is thus indicative of a protein’s binding site. However, iCLIP libraries most likely contain a mixed population of cDNAs. The position of the crosslinked peptide can cause a premature termination of cDNAs (truncated cDNAs), and computational analyses indicated that truncated cDNAs represent 80–95% of cDNAs in the analysed iCLIP libraries ( Figure 1a) ( Sugimoto *et al*., 2012). However, for the remaining cDNAs, truncation will not take place (read-through cDNAs) and thus their sequence will encompass a full RNA fragment up to the point of RNase cleavage.

In iCLIP, the start of the truncated cDNAs should be equivalent to the position of the crosslink sites. This also applies to related techniques that rely on analysis of truncated cDNAs, including among others the FAST-iCLIP, CITS-CLIP, BrdU-CLIP, irCLIP, eCLIP and miCLIP, as reviewed recently ( Lee & Ule, 2018). Using these start positions for the RNA-binding–site assignment provides high-resolution RNA-binding information ( Haberman *et al*., 2017). However, understanding the characteristics of read-through cDNAs in iCLIP is essential, since their presence could erroneously shift the boundaries of predicted RNA-binding sites to positions upstream of their true binding sites.

## Methods

### Read–through marking protocol

The modified iCLIP protocol is based on the previously described protocol with modifications that enable the definition of read–through cDNAs ( Huppertz *et al.*, 2014). HEK293 cells were crosslinked with 0.15 mJ/cm ^2^ 254 nm UV light. The cell lysate was prepared and the immunoprecipitation performed as previously described ( Haberman *et al.*, 2017). The mouse monoclonal BB7 serum anti-PTBP1 (a gift from C. Smith, available from ATCC, catalogue number CRL-2501) was used for all PTBP1 immunoprecipitations. After the first round of washes, the samples proceeded through 3’-adapter addition, an additional phosphorylation (0.2 µl PNK, 0.4 µl cold ATP (1 mM), 0.4 µl 10x PNK buffer, 3 µl water) and a 5’-marker ligation (6 µl water, 5 µl 4X ligation buffer, 2 µl RNA ligase, 1 µl RNasin, 2 µl 5’-marker (100 µM), 4 µl PEG400). The sequence of the 5’-marker is CAGUCCGACGAUC, which corresponds to the Illumina short RNA 5’-Adapter (RA5), part #15013205; this sequence is not complementary to the primers used for the amplification of the iCLIP cDNA library ( Huppertz *et al.*, 2014).

### Mapping and computational analysis of iCLIP data

The scripts used for the analyses in this paper are available in a fully documented format at the GitHub repository ( https://github.com/jernejule/non-coinciding_cDNA_starts).

Before mapping the cDNAs, we converted the FASTQ sequences into two FASTA format groups, based on presence of the sequence of the 5’-marker, CAGUCCGACGAUC, at the start of the read. Reads containing the 5’-marker sequence were marked as ‘read-through’ group. The 5’-marker sequence was then removed from further analysis and processed with the same pipeline as the remaining group of reads. To map the PTBP1 data to the genome, we used the UCSC hg19/GRCh37 genome assembly, and to map the eIF4A3 data to the transcriptome, we compiled a set of representative mRNA sequences from BioMart Ensembl Genes 79, for which we used the longest mRNA sequence available for each gene. We mapped both eIF4A3 and PTBP1 with the Bowtie2 version 2.1 alignment software, allowing two mismatches, analysed as previously described ( Haberman *et al.*, 2017).

Unique molecular identifiers (UMIs) were used to remove cDNAs that are a product of PCR amplification ( Haberman *et al.*, 2017). Thus, we quantified the number of unique cDNAs for the PTBP1 (EMBL-EBi accession number, E-MTAB-6927) and the pre-existing eIF4A3 dataset (EMBL-EBi accession number, E-MTAB-3618) after collapsing cDNAs with the same UMI and the same starting position to a single cDNA.

Prior to mapping the iCLIP data, we removed the UMIs and trimmed the 3’-Solexa adapter sequence using the FASTX-Toolkit 0.0.13 adapter removal software. For both the PTBP1 and the eIF4A3 iCLIP datasets, the cDNAs were mapped, and the Weblogo of sequence composition of genomic sequence around their starts was analysed as previously described ( Haberman *et al.*, 2017).

## Results

To examine the characteristics of read-through cDNAs that are present in iCLIP cDNA libraries, we introduced an additional RNA ligation reaction that adds an oligonucleotide to the 5’-end of the RNA fragments (5’-marker; Figure 1a, step 3b). Subsequently, only the read-through cDNAs contain the sequence of the new 5’-marker ( Figure 1a, step 8). This read-through marking protocol differs from the traditional CLIP protocol, which ligates the 5’-adapter to the RNA fragments ( Ule *et al*., 2005). In CLIP, the 5’-adapter contains a sequence that is complementary to the 5’-PCR primer, and therefore only read-through cDNAs that contain this 5’-adapter are amplified. Here, the 5’-marker is not complementary to any PCR primer and is thus not required for amplification of the cDNAs but instead becomes part of the sequenced read.

Using this modified iCLIP protocol, we produced datasets for PTBP1 and eIF4A3 ( Supplementary Figure 1). In the PTBP1 iCLIP dataset, 3.4% of the resulting reads contained the 5’-marker at their start site, while this was only the case for 0.2% of the reads for eIF4A3 ( Figure 1b, d). For the 5’-marker-containing reads, we can be confident that they are a product of read-through reverse transcription. The nucleotide composition at the start sites of these read-through cDNAs is strikingly different from the remaining cDNAs ( Figure 1c, e). The read-through cDNAs most often contain adenosine as their first nucleotide ( Figure 1c, e, at position 1), while the remaining cDNAs show an enrichment of thymidines ( Figure 1b, d, at position 1). Since the efficiency of the 5’-marker ligation is unknown, some read-through cDNAs are likely to be present in the remaining pool of cDNAs. However, the strikingly different nucleotide composition at the starts of the remaining cDNAs indicates that truncated cDNAs strongly predominate this pool.

Given that the start sites of read-through cDNAs mark the position of RNase I cleavage, it is likely that the enrichment of adenosines at this position reflects the sequence preference of RNase I, which resembles the one seen for RNase cleavage sites at the ends of cDNAs ( Haberman *et al*., 2017). By contrast, the uridine preference at the start sites of the remaining cDNAs might reflect the preference of UV-C crosslinking at uridines, as well as the binding preference of the studied RNA-binding proteins, especially PTBP1, which is known to bind U-rich motifs ( Haberman *et al*., 2017; Sugimoto *et al*., 2012).

## Conclusion

In conclusion, we established an iCLIP read-through marking approach, which ligates an additional 5’-marker to the purified RNA fragments, to examine the sequence characteristics at the start sites of read-through cDNAs as part of the iCLIP protocol. By comparing the sequence composition at the start sites of read-through cDNAs and the remaining cDNAs of CLIP libraries of selected proteins, one can more confidently define RNA-binding sites by excluding cDNAs with read-through bias at their start sites. The approach can be applied to any other method that relies on analysis of cDNAs that truncate at specific features within.

## Data availability

The PTBP1 iCLIP newly generated for this manuscript, is available from ArrayExpress, accession number E-MTAB-6927: http://identifiers.org/arrayexpress/E-MTAB-6927.

The eIF4A3 dataset produced by the read-through marking method, but published for the purpose of other analyses ( Haberman *et al.*, 2017), is available from ArrayExpress, accession number E-MTAB-3618: http://identifiers.org/arrayexpress/E-MTAB-3618.

## Software availability

**Source code available from:** https://github.com/jernejule/non-coinciding_cDNA_starts.

**License:** Creative Commons Attribution 4.0.

**Archived code at time of publication:** https://doi.org/10.5281/zenodo.213267 ( nebo56, 2018).

## Supplementary material

**Supplementary Figure 1. Quality control of the PTBP1 iCLIP experiment.** (a) Table summarising the number of cDNAs in all experiments used in this study. (b) Native acrylamide gel showing PTBP1 iCLIP PCR products that were amplified from medium (M) and high (H) cDNA size ranges. During cDNA library preparation for the modified PTBP1 iCLIP to detect ‘read-through cDNAs’, two size ranges of cDNAs were isolated from the denaturing acrylamide gel according to the recommended protocol, yielding cDNAs of approximately 90-190 nt. Since primer sequences add 52 nt to the cDNA size, sequences complementary to the iCLIP RNAs were approximately 40-140 nt. After amplification, 170-270 nt PCR products were obtained, including 128 nt of Illumina primer and iCLIP barcodes, corresponding to an approximate size range of mappable sequenced cDNAs of 40-140 nt (not shown).
