# Supplementary material for: ‘Read–through marking’ reveals differential nucleotide composition of read-through and truncated cDNAs in iCLIP
Source: Wellcome Open Res. 2018 Jun 22;3:77. [Version 1] doi: 10.12688/wellcomeopenres.14663.1 (PMC6051193; doi:10.12688/wellcomeopenres.14663.1)
Supplement: Peer review history

# Peer review: Referee response for version 1

**DOI:** 10.21956/wellcomeopenres.15965.r33400

**Published:** 2018-07-25

**Authors:** Grzegorz Kudla

**Affiliations:** 1 MRC Human Genetics Unit, Institute of Genetics and Molecular Medicine, University of Edinburgh, Edinburgh, UK

In this report, Huppertz *et al. *aim to characterize the 5' ends of reads in iCLIP experiments. This is useful because 5' ends of reads are commonly interpreted as nucleotide-resolution protein-RNA crosslinking sites in iCLIP and derived methods. The authors introduce an additional 5' marker ligation step in the experimental protocol that allows them to distinguish truncated reads from nontruncated reads based on a single sequencing dataset.

 As the authors and the other reviewers pointed out, the method only identifies a subset of nontruncated reads, because of the limited efficiency of the 5' marker ligation step. Thus, the results don't answer the question what proportion of reads are truncated - but they show that the sequence around the 5' ends differs between truncated and nontruncated reads. This finding can potentially help filter out nontruncated reads based on their 5' sequence signature, to increase the specificity of crosslinking site identification.

 Minor comments:

 1. In principle, similar conclusions could have been reached by analysing the 5' ends of reads produced by HITS-CLIP and other methods that include a 5' linker ligation step. It would be interesting to compare the results of this experiment with existing HITS-CLIP data.

 2. Does the sequence signature around the 5' end of nontruncated reads contain enough information to identify putative nontruncated reads even in the absence of 5' marker ligation?

 3. I had been under the impression that iCLIP, by definition, includes a cDNA circularization step. From the methods and Figure 1, it is not clear whether the present protocol includes cDNA circularization. Figure 1 instead mentions a "ligation od adapter to the start of cDNAs". This would need to be clarified.

I have read this submission. I believe that I have an appropriate level of expertise to confirm that it is of an acceptable scientific standard.

# Peer review: Referee response for version 1

**DOI:** 10.21956/wellcomeopenres.15965.r33401

**Published:** 2018-07-23

**Authors:** Jeremy R. Sanford, Andrew Wallace, Julia Philipp

**Affiliations:** 1 Department of Molecular, Cellular and Developmental Biology, University of California, Santa Cruz, Santa Cruz, CA, USA

This research report by Huppertz *et al.* provides a subtle but important innovation on the iCLIP protocol, which is a workhorse for labs interested in post-transcriptional regulation of gene expression by RNA binding proteins. iCLIP exploits the tendency of reverse transcriptase to terminate cDNA synthesis at residual peptide-RNA adducts. In these cases, the 3’ end of the cDNA can be interpreted as a single nucleotide resolution read out of protein-RNA interactions. However, the initial CLIP and HITS-CLIP papers, which required a 5’linker for library amplification, established that some fraction of reverse transcription events are able to read through the crosslinking site. It is possible that the read through rates, if substantial, could complicate the interpretation of iCLIP data.

 The authors address the problem of crosslinking site read through by ligating an RNA oligo to the 5’end of RNA fragments isolated by CLIP. These “marked” RNAs are processed via the standard iCLIP pipeline involving cDNA synthesis and circularization. Because the RNA marker is not required for amplification of the library, any reads containing this sequence must arise from read through. Using antibodies for two different RBPs, the authors demonstrate sequences at RT stop sites  versus read through sites are distinct. They also determine that a vast majority (>97%) of reads correspond to truncated cDNAs.

 The authors also identify an important caveat to their work- the quantification of read through cDNAs depends on the ligation efficiency of the 5’RNA oligo. In other words, it is possible that the truncated cDNAs are contaminated with unligated readthrough products. One possible improvement could be to perform an affinity selection of the ligated RNA products, using the 5’ oligo as a handle, prior to cDNA synthesis. In this case, all of the RNA molecules would contain a marker. Any reads lacking the marker sequences could only arise from truncation. We also suggest the authors highlight the extent to which their truncation percentages could vary with ligation efficiency.

 In summary, this is a technically sound experiment with literal interpretations of the data. The authors include sufficient experimental detail to reproduce the study.

 Minor point:

 In the results section, when describing the nucleotide composition at the first position of the read through and truncated (enrichment of A vs T, respectively), the authors identify the wrong panels. Figure 1b,d correspond to read through and c,e correspond to the truncated cDNA:

We have read this submission. We believe that we have an appropriate level of expertise to confirm that it is of an acceptable scientific standard.

# Peer review: Referee response for version 1

**DOI:** 10.21956/wellcomeopenres.15965.r33398

**Published:** 2018-07-12

**Authors:** Donald Rio, Qingqing Wang

**Affiliations:** 1 Department of Moelcular & Cell Biology, University of California, Berkeley, Berkeley, CA, 94720-3204, USA; 2 University of California, Berkeley, Berkeley, CA, USA

Huppert and Haberman et al. presenta revised version of the widely used iCLIP protocol called “read-through marking” that is able to differentiate read-through cDNAs from the truncated ones that result from the stoppage that occurs when the reverse transcriptase encounters the covalent adduct of RBP-RNA complex at the site of UV-crosslinking site during reverse transcription in the process of making the cDNA library. This method involves the ligation of a 5-end oligonucleotide to the RNA fragments and thus distinctively marks the cDNAs that experience read-through during reverse transcription. Overall, this method is a valuable addition to the CLIP technology and will contribute to increasing the resolution of RBP binding site detection by workers in the field. Since the invention of the original HITS-CLIP and iCLIP methods, that offer detection of the exact RBP binding site and RNA-protein crosslinking site in the target RNAs at single nucleotide resolution, multiple variations of the technology have been developed, such as fast-iCLIP, irCLIP and eCLIP. However, the majority of coordinated computational analysis tools and mapping pipelines for these technologies do not utilize the abundant information provided by these technologies that give the precise location of RBP-RNA binding. This is largely due to the fact that a (variable, but probably significant) portion of the resulting cDNAs are read-through events and do not record the exact crosslinking site of RBPs to the RNA target. As a result, computational analyses suffer from noise introduced by the read-through events that are almost impossible to differentiate from the actual truncated cDNAs. Huppert and Haberman et al., on the other hand, provide an avenue to solve this problem using a straightforward experimental measurement from the technology itself and the data presented in the manuscript strongly support the validity of their new method. We believe the field will benefit greatly from this method and analytical tool, especially in providing increased precision  and detection of RBP binding sites.

 We only have minor comments about the manuscript:

  

1. Can the authors specify further about the cDNA start site identification pipeline?Is any peak calling involved? Or are the results presented in Figures 1b-e coming from analyzing the raw cDNA start sites from all cDNA fragments?

 

1. Can the authors comment on situations when the reverse transcriptase does not truncate exactly at the crosslinking site, but instead truncates after the crosslinking site, but before reaching the 5’-end of the RNA fragment, in which case this modified analysis software will not be able to catch these events?

We have read this submission. We believe that we have an appropriate level of expertise to confirm that it is of an acceptable scientific standard.

# Peer review: Referee response for version 1

**DOI:** 10.21956/wellcomeopenres.15965.r33399

**Published:** 2018-06-28

**Authors:** Sander Granneman

**Affiliations:** 1 Institute of Structural and Molecular Biology, Centre for Synthetic and Systems Biology (SynthSys), University of Edinburgh, Edinburgh, UK

Here the authors describe an approach to quantify the degree of reverse transcriptase read-through during iCLIP cDNA library preparation. By ligating a specific oligonucleotide to the 5’ ends of cross-linked RNAs, they can roughly quantify the degree of read-through and determine whether the sequence characteristics of the read-through fragments are similar to the truncated cDNAs. The approach used is clever and the paper is of a good quality. I do have a few suggestions that might improve the paper:

 In the Methods section the authors mention what volume of PNK enzyme is used for the RNA ligation reactions but they do not specify units. I assume they are using NEB PNK (?) but this comes in various concentrations. I would appreciate it if the authors could also provide the number of units of enzymes in the materials and methods.

 One potential issue with the method is that it is not possible (or not easy) to determine whether all 5’ ends of the cross-linked RNAs have actually been ligated to their adapter. I agree with the authors that the sequence analysis strongly indicate that the ligation reaction went to completion. Have the authors done any optimization steps for the ligation reaction? It might be worth mentioning that this step may need to be optimized for each protein as the amount of RNA cross-linked to a protein can vary quite significantly.

 T4 PNK ligase also introduced sequence representation biases as the enzyme seems to have a preference for certain nucleotide combinations. Did the authors have test how well their adaptor ligates to RNAs with different nucleotides at 5’ ends. Most groups now routinely add random nucleotides to adapter sequences to minimize such bias (you can never completely remove it). Have the authors considered doing this? Perhaps the authors could comment on this in the manuscript.

 The authors have specifically looked at the 5’ ends of the read-through fragments for motif analysis. These results suggest that most of the RNA sequences in read-through fragments have A’s at the 5’ end, which is to be expected as the RNAs were fragmented with RNAse I. I am wondering though if it would not be also interesting to look at mutations within these read-through fragments to assess whether (a) all read-through fragments have mutations, (b) whether these primarily are deletions or substitutions and whether they cluster together. I would also be interested to see if the motifs found at or near deletions/substitutions are similar to the ones identified in the truncated cDNAs. Essentially, these analyses could answer a long-standing question about how reliably mutations are for pin-pointing the UV cross-linking site. The reason for asking this is that we sometimes observe deletions in U-rich regions and it is not always evident whether these deletions are the result of over cross-linking the cells (RNA damage) or whether these are actual UV-cross-linking sites.

 Because the read-through fragments may have multiple mutations, they may not map accurately to the reference sequence. Can the authors comment on how many of the read-through reads did not or poorly align to the reference sequence?

I have read this submission. I believe that I have an appropriate level of expertise to confirm that it is of an acceptable scientific standard.

# Response

**Published:** 2018-07-09

**Authors:** Jernej Ule

**Affiliations:** The Francis Crick Institute, UK

We thank the reviewer for the excellent suggestions, and we will provide a complete response along with the revised manuscript once we receive further reviews. For now, I would just like to clarify that this method doesn't work under the assumption that 5’ ends have been efficiently ligated to their adapter. In fact, more likely the opposite is true, since the 5' ends of CLIP RNAs can often be buried in the protein binding pocket, and can form RNA structures that make them inaccessible to PNK phosphorylation and ligation. Thus, we assume that the 5' ligation efficiency is probably below 50%. It would be difficult to increase it much further due to the variable accessibility of RNA fragments.

Importantly, high ligation efficiency is not needed for the analyses used in the manuscript, since we focus primarily on those reads that have the adapter - and here we can be confident that the ligation worked, so these certainly are readthrough cDNAs. The purpose of our method is to identify the sequence characteristics of the readthrough RNAs within a standard iCLIP experiment, which maximises the recovery of RNA fragments by amplyfying them regardless of whether 5' marker has been ligated or not.

A more thorough response will follow later.
